# Development of Water-in-Oil Emulsions as Delivery Vehicles and Testing with a Natural Antimicrobial Extract

**DOI:** 10.3390/molecules25092105

**Published:** 2020-04-30

**Authors:** Giovana Colucci, Arantzazu Santamaria-Echart, Samara C. Silva, Isabel P. M. Fernandes, Caroline C. Sipoli, Maria F. Barreiro

**Affiliations:** 1 Mountain Research Center (CIMO), Polytechnic Institute of Bragança, Santa Apolónia Campus, 5300-253 Bragança, Portugal; giovana.colucci@ipb.pt (G.C.); asantamaria@ipb.pt (A.S.-E.); samaras@ipb.pt (S.C.S.); 2 Department of Chemical Engineering, Federal University of Technology (UTFPR)–Paraná, 86812-460 Apucarana, Brazil; carolinesipoli@utfpr.edu.br; 3 Laboratory of Separation and Reaction Engineering—Laboratory of Catalysis and Materials (LSRE/LCM) Department of Chemical Engineering, Faculty of Engineering University of Porto, Dr. Roberto Frias, S/N, 4200-465 Porto, Portugal

**Keywords:** emulsions, water-in-oil, cinnamon extract, sweet almond oil, hydrophilic extracts, natural compounds

## Abstract

Water-in-oil (W/O) emulsions have high potential for several industrial areas as delivery systems of hydrophilic compounds. In general, they are less studied than oil-in-water (O/W) systems, namely in what concerns the so-called fluid systems, partly due to problems of instability. In this context, this work aimed to produce stable W/O emulsions from a natural oil, sweet almond oil, to be further tested as vehicles of natural hydrophilic extracts, here exemplified with an aqueous cinnamon extract. Firstly, a base W/O emulsion using a high-water content (40/60, *v*/*v*) was developed by testing different mixtures of emulsifiers, namely Tween 80 combined with Span 80 or Span 85 at different contents. Among the tested systems, the one using a 54/46 (*v*/*v*) Span 80/Tween 80 mixture, and subjected to 12 high-pressure homogenizer (HPH) cycles, revealed to be stable up to 6 months, being chosen for the subsequent functionalization tests with cinnamon extract (1.25–5%; *w*/*v*; water-basis). The presence of cinnamon extract leaded to changes in the microstructure as well as in the stability. The antimicrobial and antioxidant analysis were evidenced, and a sustained behavior compatible with an extract distribution within the two phases, oil and water, in particular for the higher extract concentration, was observed.

## 1. Introduction

Emulsions are colloidal systems consisting of two liquid phases, oil and water, one of which is dispersed into the other [1,2]. Water-in-oil (W/O) emulsions consist of an aqueous phase dispersed, in the form of small droplets, into a continuous oil phase [3,4]. W/O emulsions have high potential for cosmetic, pharmaceutical, agricultural, and food industries [3,5,6,7]. For example, this type of emulsion can be used for the encapsulation of medicines, immobilizing enzymes, and loading protein drugs [5,8,9]. Its structure is suitable for the delivery of hydrophilic compounds, which, in turn, may bring different functions to the emulsified system, such as antimicrobial and antioxidant activities.

The main challenge in emulsion technology is their stability, once they are thermodynamically unstable systems [10,11,12]. However, the kinetic transition to the water/oil separated phases can be so slow that the emulsion may be considered metastable [13]. W/O emulsions generally present lower stability than the oil-in-water (O/W) counterparts, due to the high mobility of water droplets, which causes sedimentation, flocculation, or coalescence. Besides that, only steric forces are expected to stabilize this type of emulsions due to the low electrical conductivity of the oil continuous phase [14,15].

In fact, there are only a few studies dealing with liquid W/O emulsions in the literature [3,5,15,16,17]. Some of these works are focused on the production of W/O emulsions by non-conventional methods, i.e., without using mechanical processes [3,5], and both works corroborated the lack of studies in this field. Cheng and co-workers carried out the preparation of highly monodispersed W/O emulsions by membrane emulsification using Shirasu porous glass (SPG) membranes [5]. The emulsions were composed by water, polyglycerol polyricinoleate (PGPR), and kerosene, being characterized by a droplet size of 2–10 µm and a dispersion coefficient of 0.25. Ito and co-workers used the microchannel (MC) emulsification technique to prepare monodisperse W/O emulsions from olive oil [3]. The main objective was to study the effect of process parameters in emulsion formation, providing information about interfacial and microfluidic phenomena of W/O systems for food applications.

The difficulty to obtain stable fluid W/O emulsions and the need to better understand the interactions between their components at the water–oil interface was highlighted by Ushikubo et al. [15]. They evaluated the stability of emulsions formulated with different types of oils (hexadecane and soybean oil) and emulsifiers (Span 80, lecithin, and PGPR) at two W/O ratios in order to promote the development of new products based on liquid W/O emulsions. In another study, the stability of W/O emulsions was studied concerning the evaluation of the effect of adding calcium salts in the dispersed aqueous phase [16]. The system comprised the use of PGPR and sunflower oil as the emulsifier and oil phase, respectively. Regarding food applications, Yi and collaborators analyzed the effect of emulsifiers added to the aqueous phase, namely type and concentration, on lipid oxidative stability of a water-in-walnut oil emulsion with a fixed PGPR content incorporated at the oil phase [17]. The lipid oxidation is an undesirable occurrence in food due to the production of unpleasant odors and flavors.

Due to the lack of studies about W/O emulsions, comparatively with O/W emulsions, most of the studied cases focus on emulsion’s properties such as stability and droplet formation, as well as the effects of the used preparation method. The development of functionalized W/O emulsions is therefore inexistent. In addition, there is a high tendency to use PGPR as emulsifier to achieve stable W/O emulsions, since this compound is able to increase the viscosity of the medium, reducing the rate of coalescence of water droplets [16]. Indeed, most of W/O emulsions correspond to solid or semi-solid products, butter being a common example [15]. However, these forms may not be desired for certain applications, such as topical and oral applications, justifying the need to study fluid W/O systems.

In this context, the objective of this work was to produce a stable W/O emulsion system using a natural oil to be tested as a base vehicle to incorporate natural hydrophilic extracts. The sweet almond oil is well known for its moisturizing, softening, and nutritive properties, being already used in the cosmetic and pharmaceutical industries [18]. Its use as the continuous phase confer to the emulsions high-added value for topical applications. Thus, the development of stable emulsions with this natural oil gives the possibility to create different emulsion-based products, depending on the added hydrophilic compound and imparted bioactivities.

To achieve the stated objective, a systematic study was carried out in order to find the best formulation in terms of stability along storage time. For that, different base emulsions systems were prepared by varying the emulsifier composition (Span 80/Tween 80 at 54/46 and 80/20 ratios, and Span 85/Tween 80 at 80/20 ratio) at a fixed content. A W/O ratio of 40/60 (*v*/*v*) was chosen to guarantee a high-volume fraction of water, which may facilitate the incorporation of the intended hydrophilic compounds. Moreover, the effect of the used number of high-pressure homogenization cycles was evaluated. The best base formulation was used to incorporate a cinnamon (*Cinnamomum zeylanicum*) aqueous extract, a natural extract with antimicrobial properties [19,20,21]. The obtained emulsions were characterized, and compared to the base emulsion, in terms of physical appearance, typology, and microstructure, stability, and for antimicrobial and antioxidant activities.

## 2. Results and Discussion

### 2.1. Systematic Study and Base Emulsion Selection

In order to find the best base formulation and the corresponding preparation method, the prepared emulsions were analyzed at both microscopic and macroscopic scale by optical microscopy (OM) and visual inspection, respectively. The objective was to inspect for the occurrence of destabilization phenomena during storage time. The base emulsion systems were composed by water and sweet almond oil, at a W/O ratio of 40/60 (*v*/*v*), and an emulsifier mixture. The emulsifiers Span 80 (S80), Span 85 (S85) and Tween 80 (T80), as well as the emulsifiers’ mixtures were chosen based on previous works [7,22,23]. The emulsifier composition (*v*/*v*) was S80/T80 at 54/46 (HLB 9.2) and 80/20 ratios (HLB 6.4), and S85/T80 at 80/20 ratio (HLB 4.4), coded as S80/T80 54/46, S80/T80 80/20 and S85/T80 80/20, respectively. The chosen HLB range (4–9), which in the classical HLB scale covers W/O and wettability agents, was chosen to be further experimentally validated as effective stabilizers for W/O systems. The used content of emulsifier was 6% (total emulsion-basis, *v*/*v* (%)). The effect of passing the primary emulsions through a high-pressure homogenizer (HPH) using different number of cycles (12, 21, and 24) was analyzed.

#### 2.1.1. Analysis at the Microscopic Level

**Figure 1:**
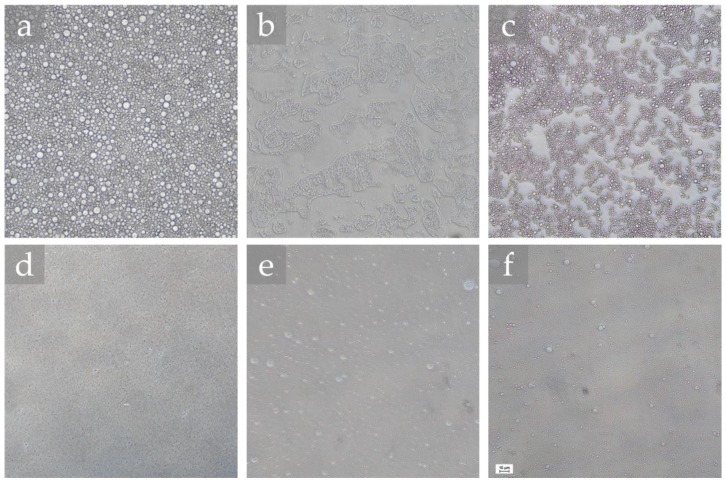
Optical microscopy of the produced 40/60 water-in-oil (W/O) emulsions. Primary emulsions: (**a**) S80/T80 54/46; (**b**) S80/T80 80/20; (**c**) S85/T80 80/20. After 12 high-pressure homogenizer HPH cycles: (**d**) S80/T80 54/46; (**e**) S80/T80 80/20; (**f**) S85/T80 80/20. Bar = 10 μm, 200× magnification.

The morphology of the base emulsions, namely the primary emulsion, and emulsions subjected to 12 HPH cycles, analyzed right after production, are shown in Figure 1. The OM images after 21 and 24 cycles can be visualized in supplementary information (Figure S1).

Analyzing the images, it was observed that, in general, all emulsions presented small and spherical droplets, evidencing a droplet size reduction, and size homogeneity increase with the applied number of HPH cycles. It should be highlighted that the emulsifiers using a ratio of 80/20 (samples S80/T80 80/20 and S85/T80 80/20, the ones with lower HLB resulted in similar morphologies after 12 HPH cycles, regardless of the used type of emulsifiers (Figure 1e,f, respectively).

Based on the OM images, droplet size range was determined in the primary emulsions and after subjecting them to 12, 21, and 24 HPH cycles, by randomly measuring 30 droplets in each acquired image. Considering the detection limit of the optical microscope, it was not possible to perform the measurements for all samples, implying that the droplet size of this emulsions was lower. The droplet size range of the analyzed emulsions is summarized in Table 1.

In general, as previously observed in the OM images, the emulsions presented small droplet sizes, corroborating the suitability of the used emulsifiers combination (S80/T80 and S85/T80) to prepare high-water content emulsion W/O systems, one of the objectives of this work. Within the observed small size ranges, it was appreciated that for the emulsifier system S80/T80, the ratio variation from 54/46 to 80/20 led to a considerable decrease in the droplet size, already in the primary emulsion. Thereafter, the reduction of the droplet size due to the effect of the applied number of HPH cycles was more effective in the S80/T80 54/46 system. In the case of using 24 cycles, an increase in the size range was observed, a fact that could be related with the occurrence of coalescence phenomena induced by an excessive number of HPH cycles, which may lead to the agglomeration of droplets as also previously reported [24]. Considering the used emulsifiers, it was observed that, for the tested ratio 80/20, a slight increase in the droplet size was observed when replacing S80 by S85.

#### 2.1.2. Analysis at the Macroscopic Level

**Figure 2:**
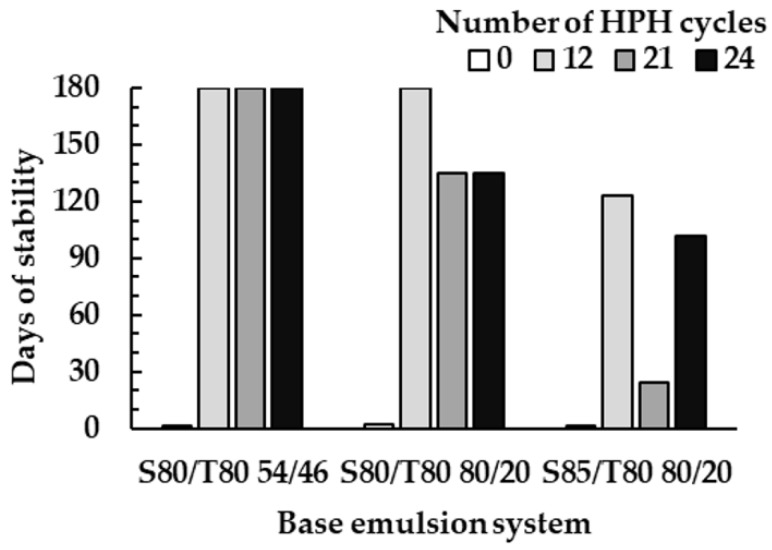
Stability over time of the base emulsions prepared using different number of HPH cycles.

The visual inspection of the emulsion systems along time was done in order to check for instability phenomena appearance at the macroscopic level. The stability of the emulsion systems prepared at a different number of HPH cycles was analyzed over 180 days and the obtained results are shown in Figure 2.

Analyzing the stability of the base emulsions, it was observed that all primary emulsions showed phase separation after a short time-period, notably, no one surpassed 2 days of stability. After applying HPH, it was observed a general tendency for a stability increase. Nevertheless, it was also observed that an excessive number of HPH cycles can revert this effect by decreasing the stability. This is in accordance with the work of Lee et al. [24] that pointed out that the application of an excessive number of HPH cycles induces destabilization phenomena, causing the emulsions to be stable for shorter periods of time. In this work, and for the analyzed conditions (12, 21, and 24 cycles), the use of 12 HPH cycles was the most suitable solution conducting to higher stability for all the three studied systems, namely 180 days for the systems S80/T80 54/46 and 80/20, and 123 days for the S85/T80 80/20 system.

Regarding the effect of the emulsifier composition, it was verified that the mixture S80/T80 54/46, the one using the emulsifier mixture with higher HLB (9.2), gives rise to higher stability. The emulsion using this emulsifier composition resulted in being stable up to 180 days of storage at room temperature, regardless of the number of applied HPH cycles. In turn, emulsions using S80/T80 80/20 (HLB 6.4) were less stable, namely when the number of applied cycles increased. Moreover, the replacement of S80 by S85 resulted in less stable emulsions.

#### 2.1.3. Base Emulsion Selection

**Figure 3:**
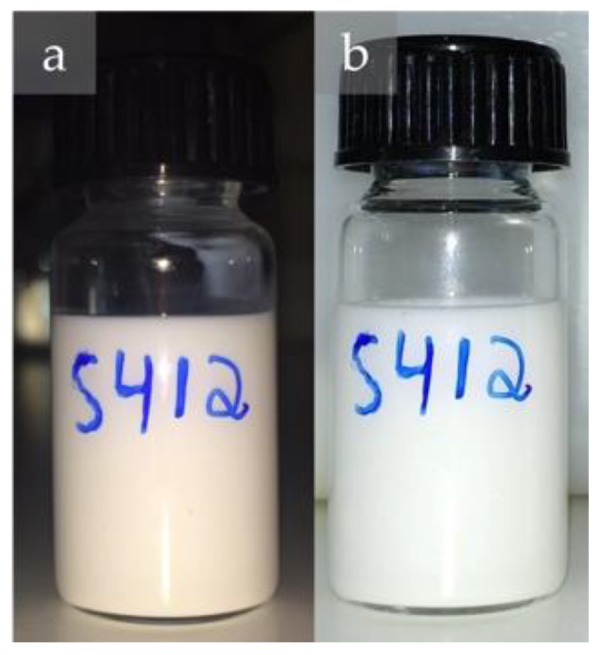
Images of system S80/T80 54/46 with 12 cycles HPH: (**a**) Fresh. (**b**) After 6 months of storage at room temperature.

At a microscopic level, all the 3 produced emulsions showed small droplet sizes (particularly after applying HPH), favoring stability due to the lowering of collision efficiency [16,25]. Concerning the stability studies analyzed at a macroscopic level, the S80/T80 54/46 sample (Figure 3) was the one presenting the higher stability (180 days), a time-frame suitable for commercial applications. Furthermore, the high stability of this emulsion, independent of the applied number of HPH cycles, offers the possibility to choose 12 HPH cycles, enhancing its competitiveness for industrial scale up, considering the lower number of cycles, short preparation time, lower energy requirements, and minor gas consumption (used to pressurized the HPH system). Thus, the formulation S80/T80 54/46 was chosen as the base formulation to proceed with the cinnamon extract incorporation studies. It possesses a high-water fraction (W/O 40/60) and is stable for a long period of time (180 days). 

### 2.2. Cinnamon Extract Loaded Emulsions

**Figure 4:**
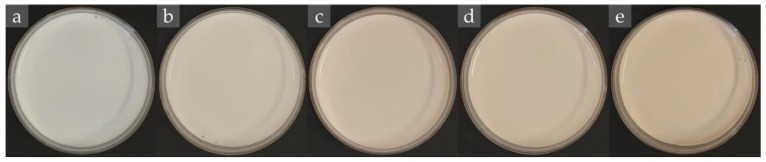
Images of W/O (**a**) base emulsion and emulsions containing (**b**) 1.25%, (**c**) 2.5%, (**d**) 3.75%, and (**e**) 5% of cinnamon extract.

The used bark extract of *Cinnamomum zeylanicum*, also known as true cinnamon or Ceylon cinnamon, presents a high content of phenolic and flavonoid compounds, including a high content of cinnamaldehyde (around 50.0%), an aromatic aldehyde that exhibits antimicrobial properties [20]. Based on the selected S80/T80 54/46 system, and the preparation method considering 12 HPH, emulsions containing cinnamon aqueous extract at different concentrations (1.25%, 2.5%, 3.75%, and 5% *w*/*v*, water-basis) were prepared and characterized concerning droplet size range (by OM), confocal laser scanning microscopy (CLSM), antimicrobial and antioxidant activity, and stability. In order to compare the results, the base emulsion was also characterized. Samples were coded as E1.25, E2.5, E3.75, and E5, reflecting the used amount of extract. The visual appearance of the produced emulsions is shown in Figure 4, where it can be observed that all of them resulted similar, observing a slight browning with the increase of extract content.

#### 2.2.1. Droplet Size Range of Loaded Emulsions

Analogously to the base emulsions, for the extract loaded ones, the droplet size range was determined by OM, and the obtained results are shown in Table 2. The images of OM employed for the determination are included in supplementary information (Figure S2).

Analyzing the results in comparison with the base emulsions, it was observed a slight increase of the droplet size due to the incorporation of the extract. For example, the base emulsion presented a droplet size under the detection limit of the microscope after 12 HPH cycles, while the corresponding loaded emulsions presented sizes ranging from 0.6 to 2.0 μm. This fact was directly attributed to the extract solubilization in the aqueous phase. Even in the primary emulsions, the loaded emulsions droplet size showed a broadening trend, indicating the lower size homogeneity derived from the incorporation of the extract. Nevertheless, it should be also noted the considerable reduction in the droplet size after the HPH treatment. These results pointed out for the effectiveness of the chosen preparation method, which leads to the obtainment of droplet size reduction, and increased size homogeneity.

#### 2.2.2. Confocal Microscopy Analysis

**Figure 5:**
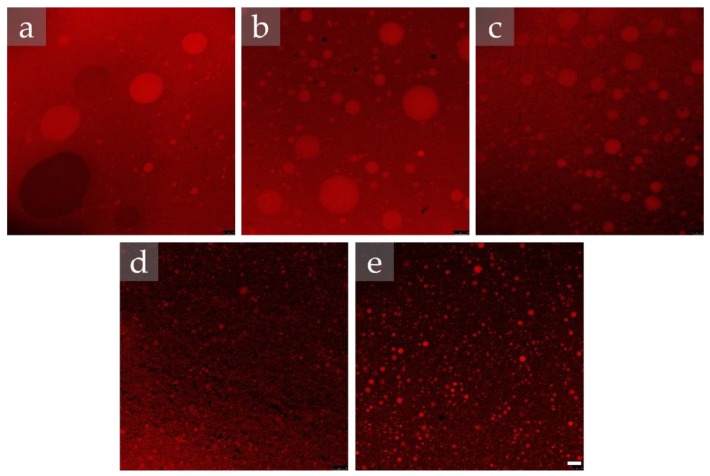
Confocal laser scanning microscopy (CLSM) images (2048 × 2048 pixels) of W/O emulsions at 40/60 ratio: (**a**) Base emulsion and emulsions containing the cinnamon extract at concentrations of: (**b**) 1.25%, (**c**) 2.5%, (**d**) 3.75%, and (**e**) 5% (*w*/*v*). Bar = 10 μm.

The microstructure of emulsions was analyzed by CLSM, using the fluorescent dye Nile Red. This dye causes the lipid background of the oil to fluoresce and the water droplets to appear as non-fluorescing black areas [26]. Figure 5 presents the images obtained from this analysis for each sample.

The prevalent continuous red coloration observed in the images is a positive indication of the formed emulsion type, water-in-oil. The water droplets are not easily perceptible due to the used magnification of 10 µm, once the water droplets might present sizes lower than 2 µm (Table 2). In fact, the deep penetration of the CLSM laser into the emulsions causes light scattering causing focus loss, being difficult to capture higher magnification images [27].

Figure 5a corresponds to the CLSM image of the base emulsion, where the presence of a fine structure can be observed. However, the continuous phase presents an inhomogeneous spatial distribution of oil, since the presence of circular red regions are noted (some areas are lighter than others). This can be due to the used W/O ratio of 40/60, which might be close to the phase inversion conditions. Furthermore, water droplets are not easily distinguished on the image due to their small size (only a fine black dotted pattern is perceptible).

Upon extract incorporation (Figure 5b–e), the appearance of black microstructures starts to be perceptible, which increased as the extract concentration in the formulation increased. The extract has a hydrophilic character, meaning that it will contribute to the increase of the back hue and the appearance of more defined black structures. Namely, at higher extract concentrations, 3.75% and 5% (Figure 5d,e, respectively), this effect is intensified, and a distribution of the extract also into the oil phase might have occurred. This is expected due to the possible saturation of the water phase and the fact that some extract compounds can show affinity with the oil phase. This distribution of the extract within both phases can impact positively in the final functional emulsion’s behavior, since it is expected that the extract present in the continuous phase become more readily available to exert the intended functionalities. The one inside the water dispersed phase with guarantee the sustainable release, and thereafter the long-lasting effect of the developed products.

#### 2.2.3. Antimicrobial Analysis

The incorporation of the cinnamon extract may give enhanced antimicrobial activity to the developed emulsions. In this way, this activity was qualitatively evaluated by the agar diffusion method with *Staphylococcus aureus* (gram+), *Escherichia coli* (gram-), and *Pseudomonas aeruginosa* (gram-) bacteria. The inhibition halos obtained for each formulation (base emulsion and emulsions added with the extract), pure components, aqueous extract solutions (AE), and positive control (kanamycin antibiotic), measured after 24 and 96 h of incubation at 37 °C are presented in Table 3.

Analyzing the obtained results, it can be noticed that the pure components, sweet almond oil, and the Span 80/Tween 80 54/46 aqueous solution (emulsifiers mixture), as well as the base emulsion, did not exhibit antimicrobial activity against all the tested bacteria during the assayed incubation period. Oppositely, the emulsions containing the cinnamon aqueous extract (E1.25 to E5) presented activity against *S. aureus* at the incubation time of 24 h (inhibition halo of 9 mm). After 96 h of incubation, the inhibition halo detected against *S. aureus* was maintained for all the tested concentrations, and activity against *E. coli* was also revealed (inhibition halo of 9 mm). This result is a direct consequence of the extract presence in the emulsion, which was protected and released throughout a period (sustained release). The prompter effect achieved at 24 h might be also related with the extract fractions present in the oil phase, which are expected to be more accessible to exert their function. Furthermore, the emulsions added with the extract presented similar inhibition zones, independently of the used extract concentration, meaning that concentration does not have a significant impact in the antimicrobial activity, for the tested times. Nevertheless, it is expected that a higher extract concentration would lead to products with higher long-lasting effect, a fact not evaluated in the present study due to the constraints associated to the used methodology.

Regarding the extract aqueous solutions (AE1.25 to AE5), the antimicrobial activity against *S. aureus* was detected after 24 h of incubation for all the tested concentrations, while only the Sample AE5 (5% extract) exhibited activity against *E.coli*, being the obtained diameters 14 and 7 mm, respectively. After 96 h of incubation, a decrease in the inhibition zones for *S. aureus* was noticed, indicating the decreased antimicrobial effect of the analyzed sample, oppositely to what happens with the loaded emulsions, where an increased effect (compatible with a sustained release) was observed.

All the tested samples (extract solutions or emulsions) did not exhibit activity against *P. aeruginosa*. This result observation might be justified by the fact that this bacterium has a double membrane involving the cell nucleus, which leads to a higher resistance against antimicrobial agents [28]. As expected, the positive control, kanamycin, presented higher inhibition zones for all the tested bacteria.

#### 2.2.4. Antioxidant Analysis

**Figure 6:**
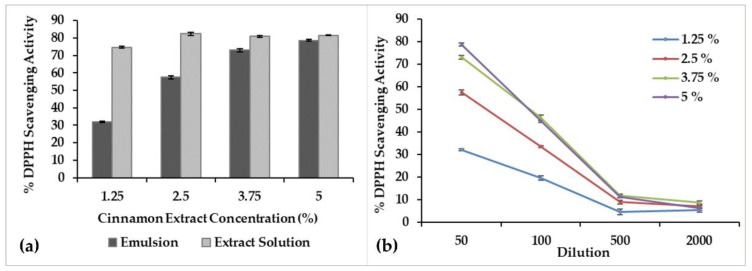
Percentage of 2,2-Diphenyl-1-picrylhydrazyl (DPPH) scavenging: (**a**) Emulsions containing cinnamon extract and the extract methanol/water solutions, for a 50× dilution. (**b**) Emulsions containing cinnamon extract at different dilutions.

The change in the absorbance produced by reduced DPPH was used to evaluate the antioxidant ability of the base emulsion, the emulsions added with cinnamon extract, and the four cinnamon extract solutions (methanol/water 80/20, *v*/*v*). The base emulsion did not present antioxidant activity, which indicates that the detected activity in the other samples is related with the presence of cinnamon extract. Figure 6 shows the obtained results, expressed as percentage of DPPH scavenging activity, for the formulations with cinnamon extract and for the extracts in solution.

From the analysis of the results presented in Figure 6a, it can be noticed that the emulsion formulations presented an increased antioxidant activity as the cinnamon extract concentration increases; however, with values smaller than the ones of the corresponding cinnamon extract solutions. At lower concentrations (1.25% and 2.5%), there is a large difference between the antioxidant activity of the emulsions and the corresponding extract solutions. This behavior is compatible with cinnamon extract being mostly protected inside the water droplets of the emulsified system, and thus not providing a prompt effect. On the other hand, for higher concentrations (3.75% and 5%), a smaller difference was noticed, which might be justified by the presence of cinnamon extract also in the external phase, as suggested by the CLSM analysis.

Figure 6b presents the comparison of the obtained results for the tested formulations using different dilutions (50, 100, 500, and 2000×). The increase in the dilution promoted the decrease of the % DPPH scavenging, i.e., the decrease of the antioxidant activity power; with values becoming closer. In addition, the performance similarity between the formulations using 3.75% and 5% is noted, as previously discussed.

#### 2.2.5. Stability Tests

**Figure 7:**
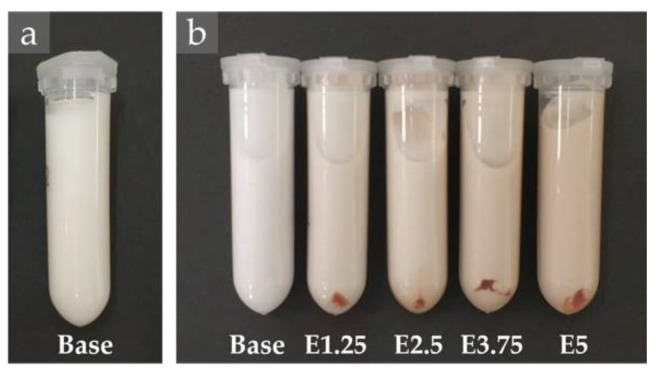
Appearance of emulsions after centrifugation at 3000 rpm for (**a**) 4 cycles and (**b**) 1 cycle.

Accelerated stability tests were performed according to centrifugation and thermal stress tests to evaluate the robustness of the produced emulsions along storage time. The appearance of the emulsions after being subjected to centrifugation stability test are shown in Figure 7. Results revealed that the base emulsion presented higher stability, once after being subjected to centrifugation at 3000 rpm (four cycles), macroscopic homogeneity was maintained, as can be observed in Figure 7a. Oppositely, for the emulsions containing the cinnamon extract, sedimentation of the extract was noticed after one centrifugation cycle, as can be appreciated in Figure 7b. The extract fractions present in the oil phase may correspond to the sedimented part (unprotected), since it was detected for all the formulations added with the extract, independently of the used concentration, but increasing with concentration increase. In addition, after the second cycle, the presence of phase separation was detected for all these formulations. Thus, the presence of cinnamon extract in the emulsion composition induced potential instability to the system.

**Figure 8:**
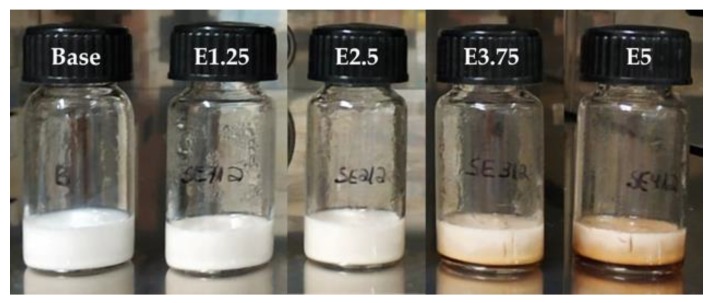
Appearance of emulsions after the thermal treatment at 80 °C.

Regarding the thermal stress tests, Figure 8 shows the appearance of the emulsions after the thermal treatment at 80 °C. All the formulations were considered highly stable to temperature, since they kept the same visual aspect up to 60 °C. At 65 °C, the emulsion added with 5% extract showed extract sedimentation and changes in the consistency. Then, at 70 °C, the system added with 3.75% extract showed a similar behavior. The thermal stress was performed until 80 °C, in which the base emulsion and the emulsions with 1.25% and 2.5% extract remained stable. These results suggest that the incorporation of high extract amounts could reduce the thermal stability of emulsions, but only noticed at high temperatures.

The observed thermal stability up to 60 °C is considered adequate for applications such as cosmetics and pharmaceuticals. For higher temperature applications, a balance between the cinnamon content and stability should be considered in view of the desired bioactivities. The antimicrobial properties were found not to be a limiting parameter, since all the loaded emulsions showed quite similar results. For the antioxidant properties, a balance should be attained, e.g., the antioxidant properties of the 3.75% and 5% loaded emulsions are proximate, with the 3.75% one showing a higher thermal stability.

## 3. Materials and Methods

### 3.1. Materials

Polyethoxylated sorbitan ester Tween 80 (T80), and sorbitan esters Span 80 (S80) and Span 85 (S85), used as emulsifiers, were purchased, respectively, from PanReac AppliChem (Barcelona, Spain), AlfaAesar (Karlsruhe, Germany) and Sigma-Aldrich (Darmstadt, Germany). The sweet almond oil was acquired from LabChem (Santo Antão do Tojal, Portugal), and the cinnamon (*Cinnamomum zeylanicum*) aqueous extract supplied by Essência D’um Segredo (Seixal, Portugal). The Nile Red was supplied by Sigma-Aldrich (Darmstadt, Germany), and Isopropyl alcohol from Merck (Darmstadt, Germany). Kanamycin sulfate (antibiotic) was purchased from Merck (Darmstadt, Germany), Mueller Hinton II Agar was purchased from Liofilchem (Roseto degli Abruzzi, Italy). For Ringer solution preparation, Sodium Chloride, Sodium Hydrogen Carbonate and Calcium Chloride 2-hydrate were purchased from Pancreac Applichem (Barcelona, Spain), Potassium Chloride was purchased from HiMedia (Mumbai, India). Methanol and 2,2-Diphenyl-1-picrylhydrazyl (DPPH) were purchased from Sigma-Aldrich (Darmstadt, Germany). All reagents were directly used without further purification. Water used in this work was distilled water.

### 3.2. Emulsions Preparation

To prepare the base system, a high-water content (W/O of 40/60, *v*/*v*), and an emulsifier content of 6% (total emulsion-basis, % (*v*/*v*)), were chosen. Three emulsifier systems were tested, namely S80:T80 54:46 (*v*/*v*), S80:T80 80:20 (*v*/*v*), and S85:T80 80:20 (*v*/*v*). The chosen compositions and contents were based on previous published works [7,22,23]. The preparation method, developed in-house, comprised, firstly, the preparation of a primary emulsion, followed by the application of successive HPH cycles (12, 21, and 24). The primary emulsion comprised the addition of the emulsifier mixture to the water phase for homogenization (10 min under stirring) followed by the addition of the oil phase and homogenization using a Unidrive X1000 Homogenizer Drive (CAT Scientific, Germany) at 11,000 rpm for 5 min. An aliquot of this primary emulsion was withdrawn for microscopic analysis. Afterwards, the primary emulsion was subjected to successive HPH cycles using an EmulsiFlex-C3 (Avestin, Canada) at 1500 bar.

To prepare the emulsions loaded with the cinnamon (*Cinnamomum zeylanicum*) aqueous extract, the extract (1.25%, 2.5%, 3.75%, and 5%; *w*/*v*; water-basis) was previously dissolved in the water phase before adding the emulsifier mixture. The following steps were as previously described for the base emulsion preparation.

### 3.3. Optical Microscopy Analysis

For microscopic images, a drop of emulsion was placed on a microscope slide and then covered with a cover slip. The used apparatus was an optical microscope NiU (Nikon microscope Eclipse Ni, Nikon Corp., Tokyo, Japan) equipped with a digital camera and NIS-Elements Documentation software. The images were made shortly after the preparation of each system, using a magnification of 200×. The images were employed for the determination of the droplet size by averaging the diameter of 30 droplets in the image. Both base emulsions and emulsions added with extract were analyzed.

### 3.4. Visual Analysis

The macroscopic appearance of the produced emulsions was periodically checked by visual inspection and photographically registered. This procedure was used for the base emulsions along a 6-month period. The samples were stored at room temperature.

### 3.5. Confocal Microscopy Analysis

Confocal Laser Scanning Microscopy (CLSM) was done using a Leica TCS-SP5 AOBS confocal microscope (Leica Microsystem Inc., Wetzlar, Germany). Emulsions were stained after their preparation by mixing 15 mL of each sample with 1 mL of Nile Red solution in isopropyl alcohol (0.1% *w*/*v*) to stain the oily phase. Then, 10 µL of the sample were placed on a concave glass slide and examined using a 40× objective. The CLSM was operated using a laser excitation wavelength of 561 nm. Only the base emulsion and emulsions added with extract were analyzed.

### 3.6. Antimicrobial Assays

The antimicrobial activity evaluation followed the agar diffusion procedure based on Kirby-Bauer method according to the ASTM E2149-01 standard [29]. The tested microorganisms were the *Pseudomonas aeruginosa* ATCC 9027, the *Escherichia coli* AATCC 10536, and the *Staphylococcus aureus* ATCC 29213. The base emulsion, the four emulsions added with the cinnamon extract, and the four extract aqueous solutions prepared at the same extract concentration as the corresponding emulsion, plus the pure sweet almond oil, the aqueous mixture of Span 80/Tween 80 54/46 (*v*/*v*), were assayed. Kanamycin solution (1 mg/mL) was used as the positive control. Briefly, each bacterium was first cultured in a nutrient broth at 37 °C for 24 h. The inoculum was diluted to 0.5 McFarland turbidity standard (1.5–3.0 CFU/mL) using sterilized Ringer solution. Then, the bacteria solution was inoculated in Mueller Hinton Agar plates, using a sterilized swab. The inoculated plates were left to dry for a short period of time. After that, a hole with 7 mm of diameter was made in the center of the plate and 100 µL of each sample placed in the hole. The plates were prepared in duplicate and incubated at 37 °C for 24 h. After this period, the diameter of the inhibition zone was measured, the incubation proceeded for a further 4 days and the diameter of the inhibition zone was measured again. The inhibition zone was determined according to the Kirby–Bauer method [30].

### 3.7. Antioxidant Assay

The antioxidant activity of the base emulsion and the four emulsions added with the cinnamon extract were assayed by evaluating the ability to scavenge 2,2-diphenyl-1-picrylhydrazyl (DPPH), a stable free radical [31,32]. The samples were diluted 50-, 100-, 500-, and 2000-fold in an 80/20 (*v*/*v*) methanol aqueous solution. To compare the results with the performance of the free extract, the same procedure was applied to solutions prepared from the same solvent mimicking the extract concentration used in the emulsions. A methanol solution was used as control. The prepared solutions (30 μL) were added with 270 μL of DPPH·methanol solution (6 × 10^−5^ mol/L) and then incubated at 37 °C for 1 h in dark conditions. The absorbance of the reaction solution was measured at 517 nm using a microplate reader (BioTek, Winooski, VT, USA). Each sample was analyzed in triplicate. The percentage of the free radical scavenging was calculated according to Equation (1):

(1) $$\%\;\mathrm{Scavenging}\;\mathrm{activity}=\frac{\mathrm{Abs}\;\left(\mathrm{control}\right)-\mathrm{Abs}\;\left(\mathrm{sample}\right)}{\mathrm{Abs}\;\left(\mathrm{control}\right)}\times 100$$

where Abs (control) and Abs (sample) are the absorbances measure for the control and the analyzed sample, respectively. 

### 3.8. Stability Tests

The base emulsion and the four emulsions with cinnamon extract were submitted to accelerated stability tests, namely, the centrifugation and thermal stress tests. The centrifugation test was performed in duplicate by using a microcentrifuge (Labogene, Copenhagen, Denmark), in which conical Eppendorf tubes containing a sample volume of 2 mL were submitted to four cycles at 3000 rpm for 30 min. After each cycle, the emulsions were visually inspected [33,34].

The thermal stress test was performed using an incubator (Raypa, Barcelona, Spain). For that, the samples, in duplicate, were successively heated along a temperature range from 25 °C to 80 °C, in which the temperature was increased 5 °C every 30 min and the emulsions visually observed at each increase [33,34].

## 4. Conclusions

In this work, a stable W/O base emulsion was prepared from a natural oil, sweet almond oil, aiming at testing the incorporation of natural hydrophilic extracts. The systematic study with the three base formulations pointed out the one using Span 80/Tween 80 at a ratio of 54/46 (*v*/*v*) as fulfilling the requirements of stability and easy preparation procedure. This system presented macroscopic and microscopic stability under storage at room temperature for six months, besides presenting the lower droplet size for 12 HPH cycles. The emulsions added with the cinnamon extract showed that the incorporation of the extract contributed to the observed changes in the droplet size, microstructure as well as in decreased stability. The CLSM analyses confirmed the water-in-oil morphology besides evidencing the microstructure change as a function of the cinnamon extract increase, namely by providing indications of the increased amount of extract in the external oil phase as the extract concentration in the formulation increased. The antimicrobial and antioxidant analysis evidenced the extract activity, once only the emulsions added with extract presented this functionality. In addition, when compared with the action of the free extract (i.e., without being protected in the emulsion), the emulsions proved to have a prolonged effect compatible with a sustained released along the time. However, the presence of extract led to a weaker stability to centrifugation, once sedimentation was perceived right after the first centrifugation cycle, a fact not observed for the base emulsion that remained stable. Regarding thermal stability, all the emulsions remained stable up to 60 °C, which can be considered an important achievement for several applications (e.g., cosmetics and pharmaceuticals). Only at higher temperatures, and for the formulations added with high extract content (3.75% and 5%), instability was observed. Overall, the results obtained herein pointed out the successful development of a stable W/O base emulsion, which might be used as a suitable delivering vehicle for hydrophilic extracts with potential use in cosmetic and pharmaceutical applications. Moreover, this study contributes for the valorization of W/O systems, by providing functional W/O emulsions based on natural compounds.

## Figures and Tables

**Table 1:** Droplet size evolution of base emulsions as a function of the applied number of HPH cycles.

Base Emulsion	Droplet Size (μm)			
	Primary Emulsion	12 Cycles	21 Cycles	24 Cycles
S80/T80 54/46	3.5–7.1	ND	ND	0.5–1.4
S80/T80 80/20	0.5–1.1	0.4–1.0	ND	ND
S85/T80 80/20	0.7–1.7	0.6–1.2	0.2–0.7	ND

ND: not determined due to the detection limit of the microscope.

**Table 2:** Droplet size range of cinnamon extract loaded emulsions.

Sample	Cinnamon Content (%, *w*/*v*)	Droplet Size (μm)	
		Primary Emulsion	12 Cycles
Base Emulsion	0	3.5–7.1	ND
E1.25	1.25	1.0–15.4	0.8–1.4
E2.5	2.50	1.5–10.0	0.8–2.0
E3.75	3.75	1.0–14.8	0.7–1.3
E5	5.00	1.0–11.3	0.6–1.6

ND: not determined due to the detection limit of the microscope.

**Table 3:** Inhibition zones achieved with the evaluated formulations through the agar diffusion test.

Sample	% Cinnamon (*w*/*v*)	Inhibition Zone ^1^ (mm)					
		After 24 h			After 96 h		
		*S. aureus*	*E. coli*	*P. aeruginosa*	*S. aureus*	*E. coli*	*P. aeruginosa*
Base Emulsion	0	-	-	-	-	-	-
E1.25	1.25	9	-	-	9	9	-
E2.5	2.5	9	-	-	9	9	-
E3.75	3.75	9	-	-	9	9	-
E5	5	9	-	-	9	9	-
AE1.25	1.25	10	-	-	9	9	-
AE2.5	2.5	10	-	-	9	9	-
AE3.75	3.75	10	-	-	10	9	-
AE5	5	14	7	-	12	9	-
Kanamycin	0	30	30	15	30	32	35
Sweet Almond Oil	0	-	-	-	-	-	-
Emulsifier mixture	0	-	-	-	-	-	-

^1^ Inhibition zone including hole with 7 mm of diameter. - means no inhibition.

## Supplementary Materials

The following are available online, Figure S1. Optical microscopy of the produced 40/60 W/O base emulsions. After 21 HPH cycles: (a) S80/T80 54/46; (b) S80/T80 80/20; (c) S85/T80 80/20. After 24 HPH cycles: (d) S80/T80 54/46; (e) S80/T80 80/20; (f) S85/T80 80/20. Bar = 10 μm, 200× magnification., Figure S2. Optical microscopy of the produced 40/60 W/O emulsions added with cinnamon extract. Primary emulsions: (a) 1.25%; (b) 2.5%; (c) 3.75%; (d) 5%. After 12 HPH cycles: (e) 1.25%; (f) 2.5%; (g) 3.75%; (h) 5%. Bar = 10 μm, 200× magnification.
