# Supplementary material for: Development of Water-in-Oil Emulsions as Delivery Vehicles and Testing with a Natural Antimicrobial Extract
Source: Molecules. 2020 Apr 30;25(9):2105. doi: 10.3390/molecules25092105 (PMC7248747; doi:10.3390/molecules25092105)
Supplement: Supplementary file 1 [file molecules-25-02105-s001.pdf]

Supplementary Material for

Development of Water-In-Oil Emulsions as Delivery Vehicles and Testing with a Natural Antimicrobial Extract

Giovana Colucci[1,2], Arantzazu Santamaria-Echart[1], Samara C. Silva[1,3], Isabel P. M. Fernandes[1,*], Caroline C. Sipoli[2] and Maria F. Barreiro[1,*]

[1] Mountain Research Center (CIMO), Polytechnic Institute of Bragança, Santa Apolónia Campus, 5300-253 Bragança, Portugal
[2] Department of Chemical Engineering, Federal University of Technology – Paraná, Apucarana, Brazil
[3] Laboratory of Separation and Reaction Engineering – Laboratory of Catalysis and Materials (LSRE/LCM) Department of Chemical Engineering, Faculty of Engineering University of Porto, Rua Dr. Roberto Frias, S/N, 4200-465 Porto, Portugal

* **Corresponding author:** e-mail: ipmf@ipb.pt
* **Corresponding author:** e-mail: barreiro@ipb.pt

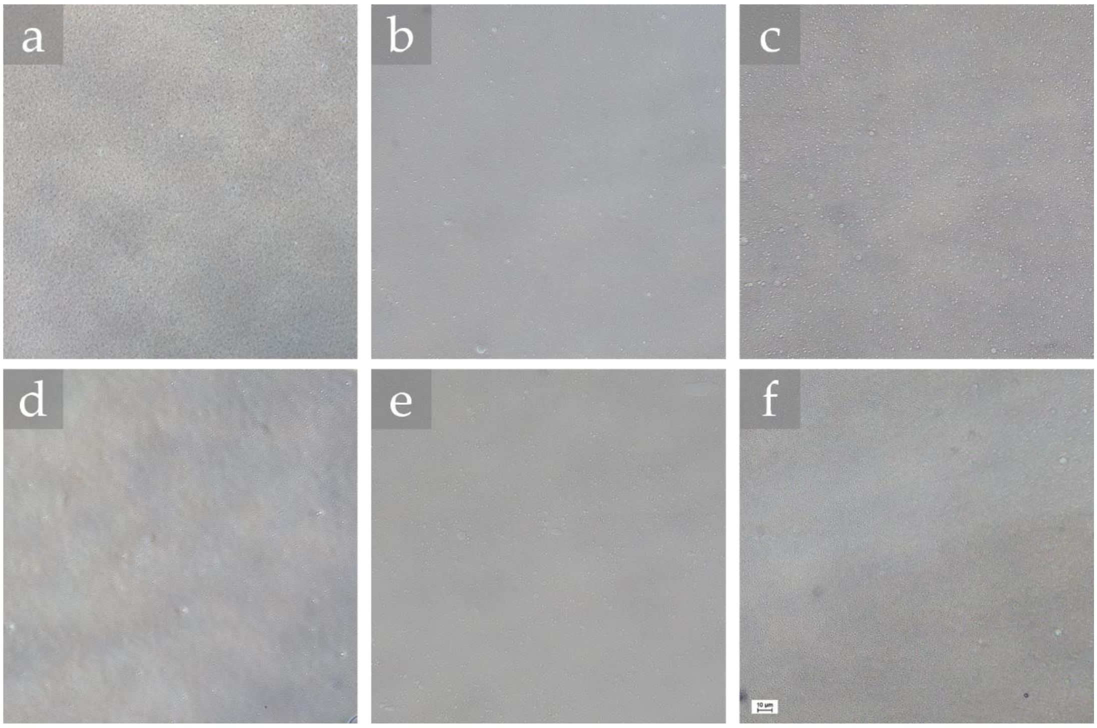

Figure S1. Optical microscopy of the produced 40/60 W/O base emulsions. After 21 HPH cycles: (**a**) S80/T80 54/46; (**b**) S80/T80 80/20; (**c**) S85/T80 80/20. After 24 HPH cycles: (**d**) S80/T80 54/46; (**e**) S80/T80 80/20; (**f**) S85/T80 80/20. Bar = 10 μm, 200× magnification.

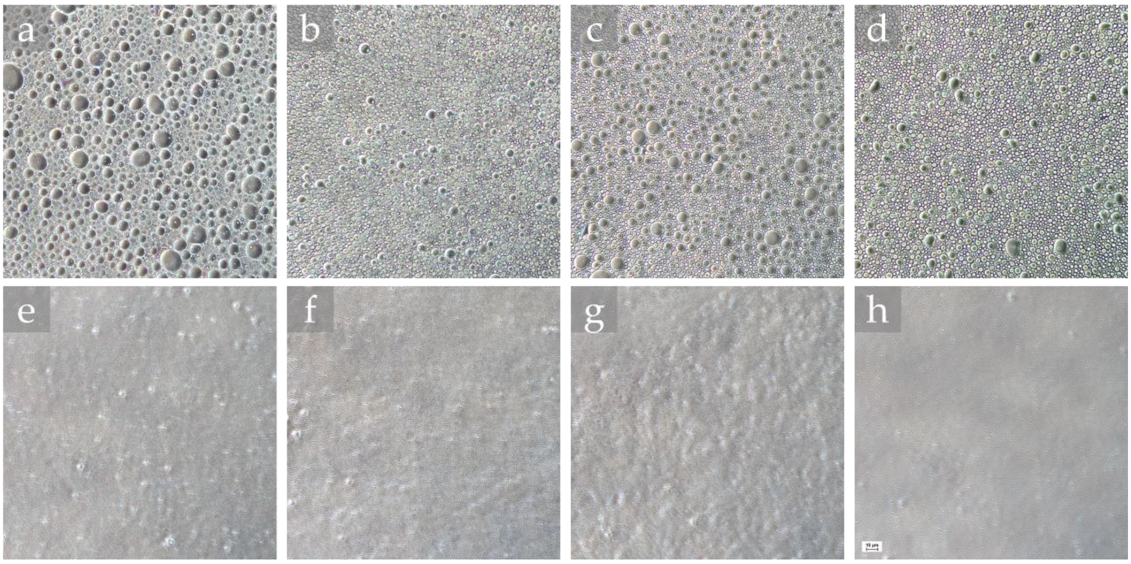

Figure S2. Optical microscopy of the produced 40/60 W/O emulsions added with cinnamon extract. Primary emulsions: (**a**) 1.25%; (**b**) 2.5%; (**c**) 3.75%; (**d**) 5%. After 12 HPH cycles: (**e**) 1.25%; (**f**) 2.5%; (**g**) 3.75%; (**h**) 5%. Bar = 10 μm, 200× magnification.
